# Probable paradoxical pyoderma gangrenosum associated with secukinumab in a patient with severe plaque psoriasis

**DOI:** 10.1016/j.jdcr.2026.07.022

**Published:** 2026-07-20

**Authors:** Carla Fernanda Genevcius, Raphael Fernando Fonseca Genevcius, Luiz Carlos da Silva, Pedro Marabini Filho, Livia Moura Tessarini Gandolfi

**Affiliations:** a Dermatology and Plastic Surgery, Clínica Genevcius, Assis, São Paulo, Brazil; b Faculdade de Medicina, Fundação Educacional do Município de Assis - FEMA, Assis, São Paulo, Brazil; c Department of Pathology, São Paulo, Brazil

**Keywords:** biologic therapy, paradoxical reaction, psoriasis, pyoderma gangrenosum, secukinumab

## Introduction

Pyoderma gangrenosum (PG) is an uncommon neutrophilic dermatosis characterized by rapidly progressive painful ulcerations and difficult diagnosis.^1^^,^^2^ Histopathologic findings are frequently nonspecific, making clinicopathologic correlation essential.^3^^,^^4^

Drug-associated PG has become increasingly recognized with the expanding use of biologic therapies.^5^ Secukinumab, an interleukin-17A inhibitor widely used for psoriasis, has rarely been associated with paradoxical PG.^6^, ^7^, ^8^, ^9^, ^10^ We report a probable case of paradoxical PG associated with secukinumab in a patient with severe plaque psoriasis and exuberant scalp involvement.

## Case report

A 40-year-old woman with a 20-year history of severe plaque psoriasis without psoriatic arthritis presented with uncontrolled disease despite previous treatment with methotrexate, narrowband ultraviolet B phototherapy, and topical corticosteroids. Baseline severity scores included Psoriasis Area Severity Index 60 and Dermatology Life Quality Index 25. At the time secukinumab was initiated, the patient was not receiving methotrexate, systemic corticosteroids, or any other systemic immunosuppressive therapy.

Secukinumab was initiated at standard psoriasis dosing (300 mg weekly during induction followed by monthly maintenance). Approximately 15 to 20 days after treatment initiation, painful inflammatory nodules developed on the gluteal region and posterior thighs. Initially interpreted as recurrent furunculosis, the lesions progressively evolved into painful ulcerations with necrotic features (Fig 1). As the patient did not initially relate the lesions to secukinumab, the drug was repeatedly administered throughout the induction phase until January, despite progressive worsening of the cutaneous lesions.

**Fig 1:**
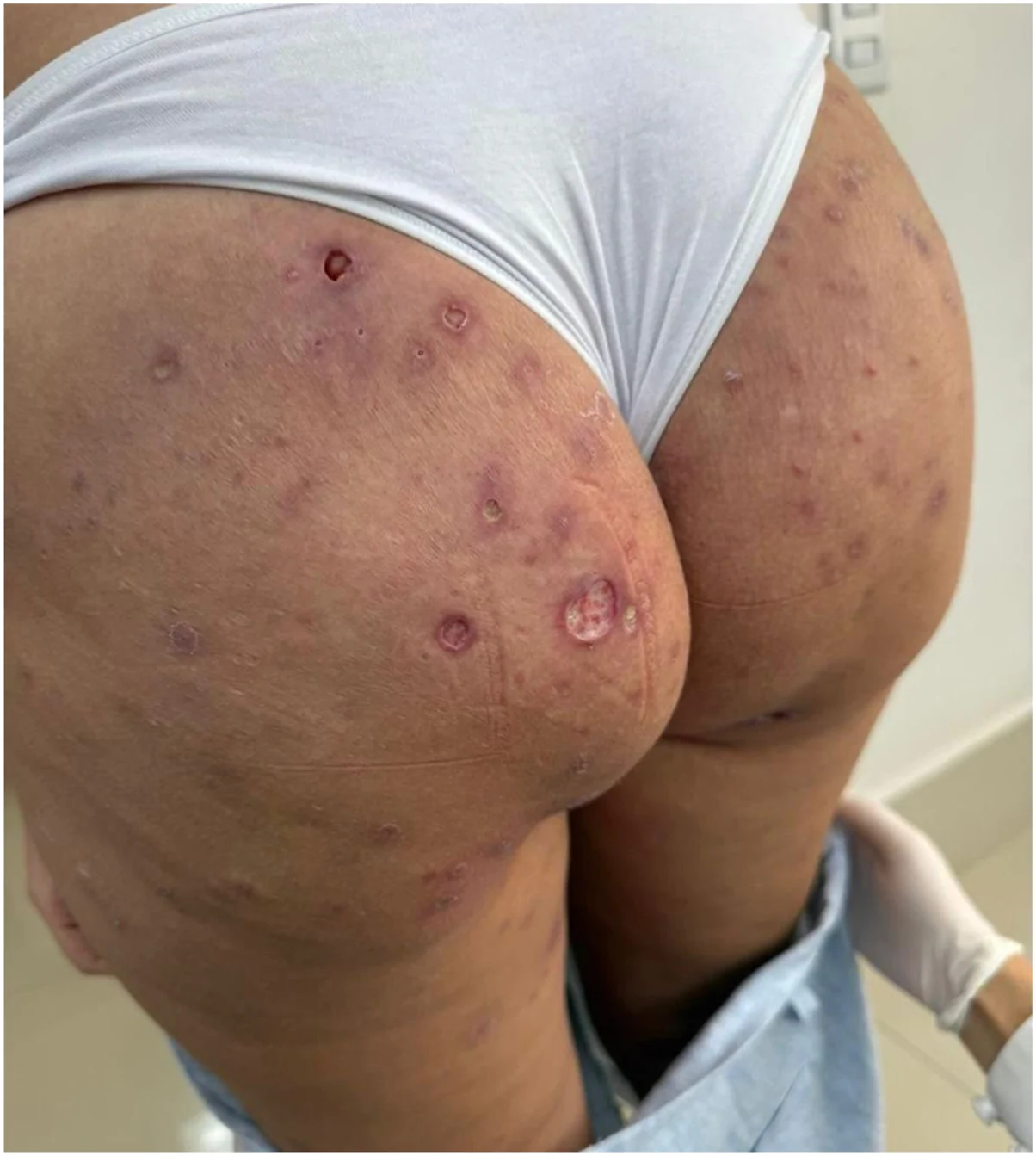
Multiple painful ulcerated papules and nodules involving the gluteal region and posterior thighs with necrotic centers and violaceous inflammatory borders.

Approximately 30 days later, rapidly progressive scalp ulcerations developed and became the predominant site of disease. Lesions exhibited irregular undermined violaceous borders, peripheral erythema, edema, purulent exudate, and severe pain, with marked impairment of daily activities (Fig 2 and 3).

**Fig 2:**
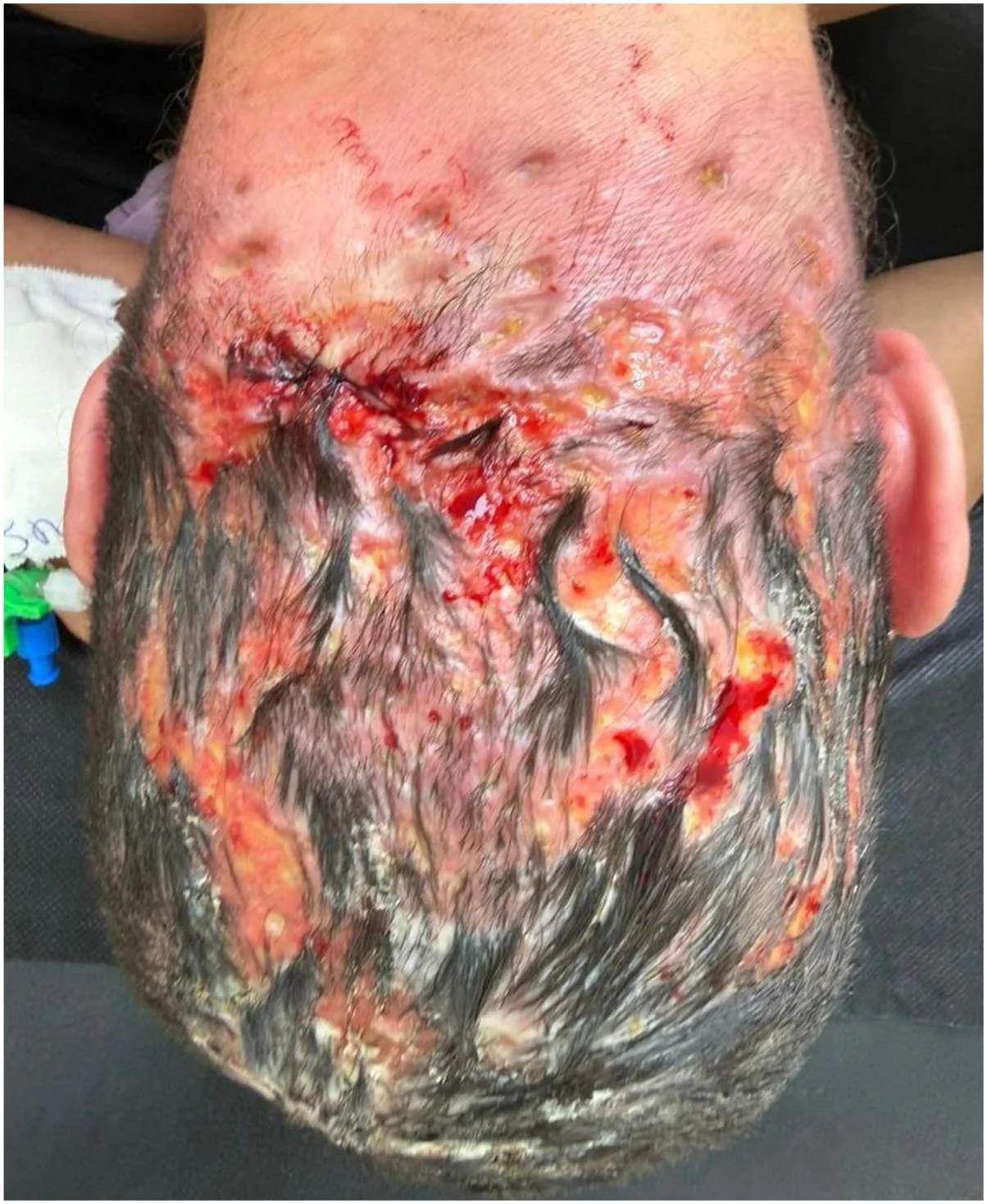
Extensive ulcerative scalp lesions with irregular undermined violaceous borders, peripheral erythema, and purulent exudate.

**Fig 3:**
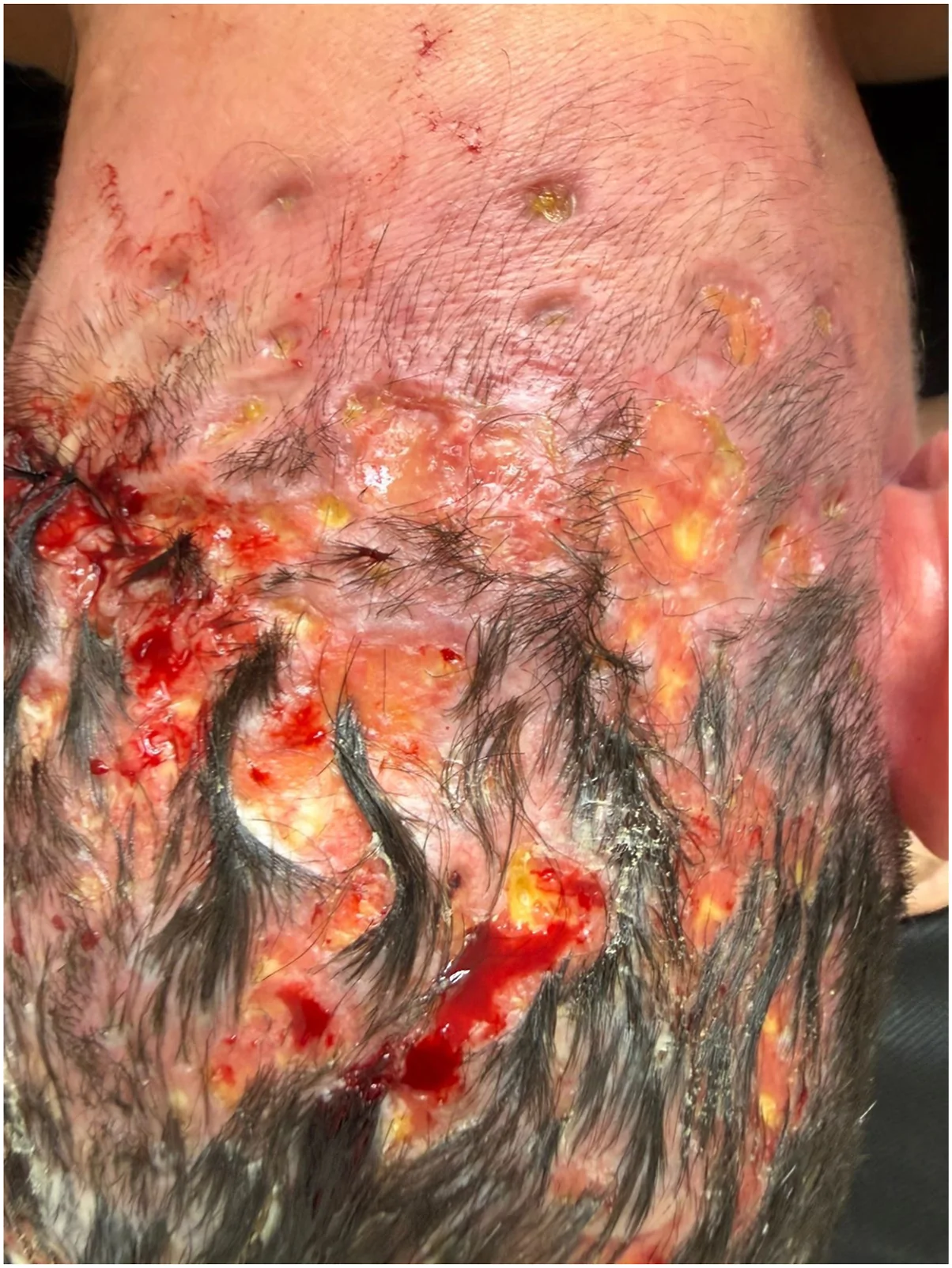
Close-up view demonstrating coalescing scalp ulcerations with superficial necrosis.

Due to extensive ulceration and concern for severe infection, the patient was hospitalized and empirically treated with intravenous vancomycin and piperacillin/tazobactam. Although repeated bacterial, fungal, and mycobacterial cultures from active lesions were consistently negative, broad-spectrum antibiotic therapy was maintained because of the severity and extent of the ulcerations. An extensive infectious workup, including serologic testing for HIV, syphilis, and viral hepatitis, was also negative. During hospitalization, repeated cleansing and debridement of the scalp ulcers resulted in marked worsening of the lesions, suggesting a pathergic phenomenon.

A biopsy specimen obtained from the active scalp ulcer edge demonstrated epidermal ulceration with fibrinoneutrophilic crust formation and focal abscesses. The dermis showed vascular proliferation and predominantly lymphocytic inflammatory infiltrate, with septal and lobular lymphoplasmacytic infiltrate in the subcutaneous tissue (Fig 4). No biopsy was performed on the gluteal or thigh lesions because the scalp ulcers became the predominant and most active site of disease.

**Fig 4:**
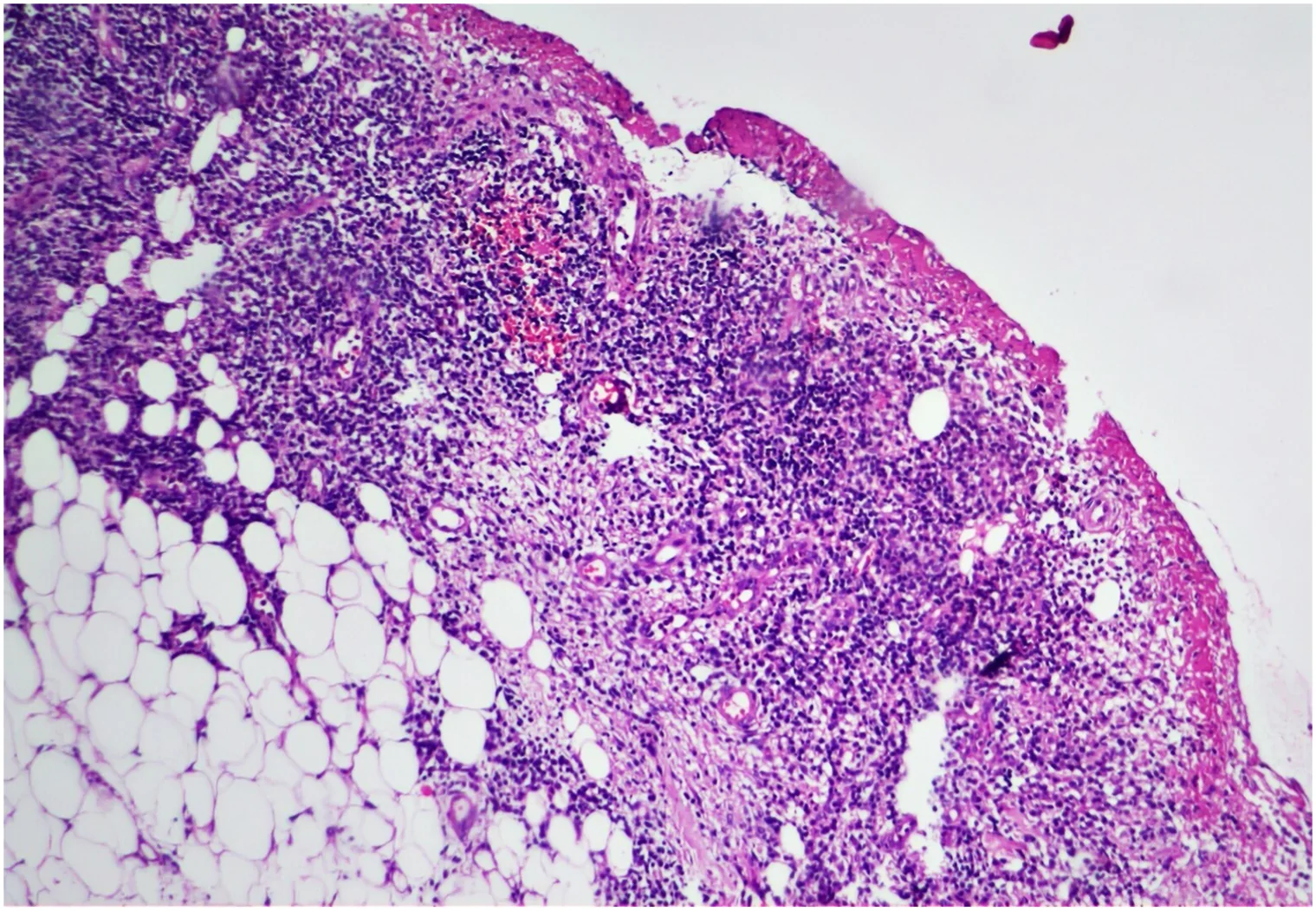
Post-treatment aspect showing resolution of inflammation and residual cicatricial alopecia.

Although dense dermal neutrophilic infiltrates were not prominent, the diagnosis of probable PG was supported by clinicopathologic correlation, fulfillment of multiple Delphi minor criteria, and a PARACELSUS score of 20 points, including rapid disease progression, exclusion of relevant differential diagnoses, violaceous wound borders, severe pain, pathergy, irregular ulcer morphology, response to immunosuppressive therapy, compatible histopathologic findings, lower extremity involvement, and underlying inflammatory disease. Because the major Delphi criterion was not fully met, the diagnosis was considered probable rather than definitive PG. The temporal association with secukinumab supported a probable paradoxical drug-induced mechanism.

Because of the initial concern for severe infection, systemic corticosteroids were withheld during hospitalization. After repeated negative cultures and exclusion of infectious etiologies, secukinumab was discontinued, and systemic corticosteroid therapy was initiated 20 days after hospital admission, resulting in dramatic pain relief within 48 hours. One week later, cyclosporine was introduced at 4 mg/kg/day, combined with local wound care, topical corticosteroids, and adjunctive hyperbaric oxygen therapy. Progressive reduction in inflammation and ulcer size was observed within the first month.

Cyclosporine was maintained for 6 months with gradual tapering according to clinical response. Most lesions achieved complete re-epithelialization after approximately 6 months, although residual cicatricial alopecia persisted (Fig 5). Psoriasis remained clinically stable with topical therapy alone, and no recurrence has been observed during follow-up.

**Fig 5:**
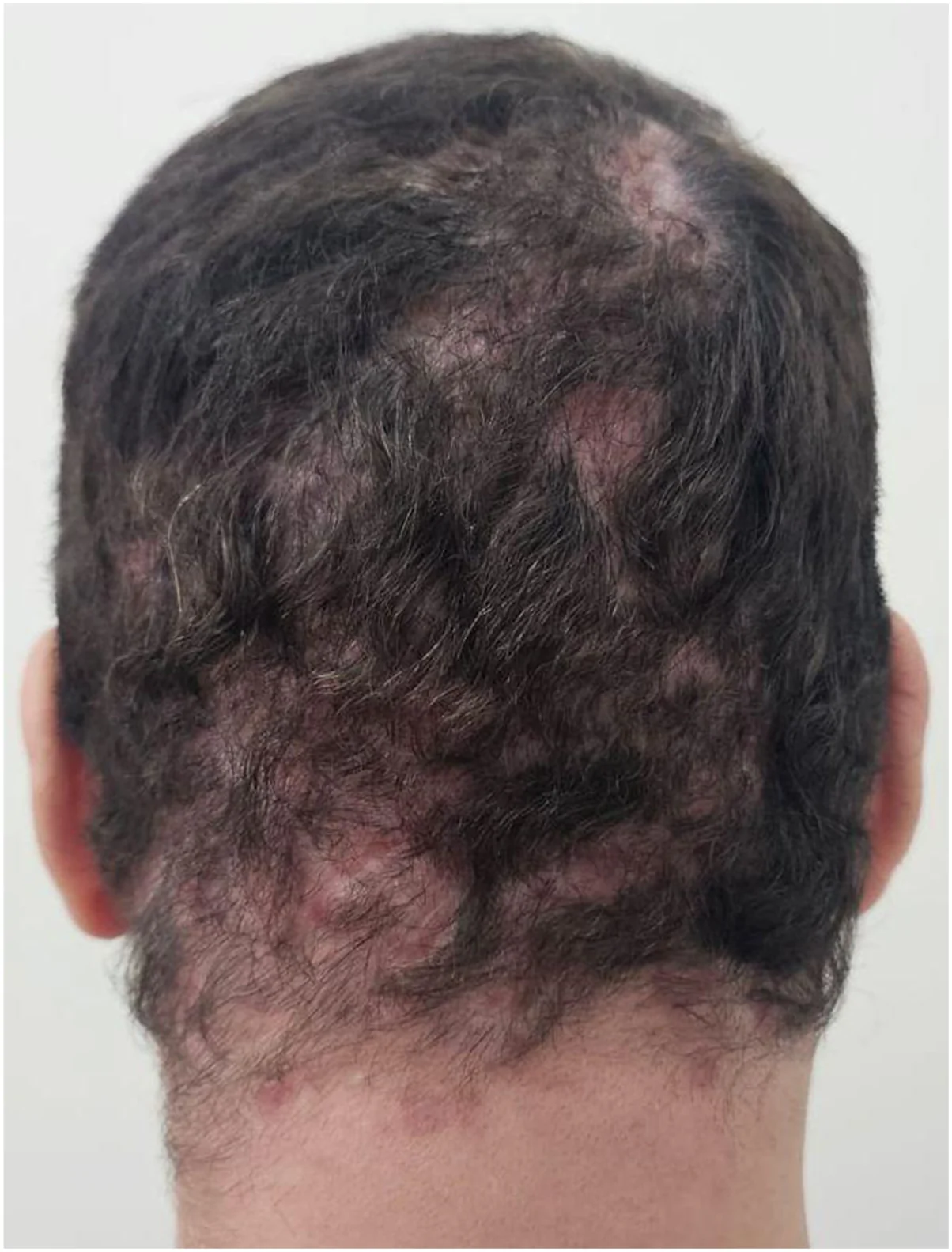
Histopathologic findings demonstrating epidermal ulceration with fibrinoneutrophilic crust and predominantly lymphocytic inflammatory infiltrate (hematoxylin-eosin stain; original magnification ×40).

## Discussion

PG remains a diagnostic challenge because histopathologic findings may vary according to lesion stage, biopsy site, chronicity, and previous treatment exposure.^3^^,^^4^ Although a neutrophilic infiltrate represents the major Delphi criterion, late ulcerative lesions may demonstrate mixed or predominantly lymphocytic inflammation, reinforcing that PG is fundamentally a clinicopathologic diagnosis.^4^

Drug-associated PG has gained increasing recognition in recent years, particularly in the context of biologic therapies capable of disrupting cytokine balance and neutrophil homeostasis.^5^ Biologic agents, especially tumor necrosis factor and IL-17 inhibitors, have been associated with paradoxical inflammatory reactions.^6^^,^^7^ Although secukinumab has demonstrated substantial efficacy in psoriasis treatment, paradoxical neutrophilic dermatoses including PG have rarely been reported.^6^, ^7^, ^8^, ^9^, ^10^ Recognition of this phenomenon is essential, as it may be misinterpreted as disease progression, infection, or treatment failure.

Our patient fulfilled several Delphi minor criteria, including exclusion of infection, rapidly progressive painful ulceration, violaceous undermined borders, underlying inflammatory disease, and significant improvement following immunosuppressive therapy. Importantly, the initial gluteal and posterior thigh lesions were interpreted as recurrent furunculosis before progression to more characteristic ulcerative lesions, illustrating a frequent diagnostic pitfall in PG.

The pathophysiology of paradoxical PG associated with IL-17 inhibition remains incompletely understood. One proposed mechanism involves cytokine imbalance following IL-17 blockade, leading to compensatory activation of inflammatory pathways and neutrophil dysregulation.^3^^,^^10^ Interestingly, IL-17 inhibitors have also been reported as therapeutic options for refractory PG,^6^ highlighting the paradoxical and bidirectional role of cytokine modulation.

Most previously reported cases of secukinumab-associated PG involved the lower extremities and demonstrated classic neutrophilic infiltrates.^7^, ^8^, ^9^, ^10^ In contrast, our case was notable for exuberant scalp involvement, multifocal progression, and less characteristic histopathologic findings. Such an unusual clinical presentation may suggest that paradoxical drug-induced PG can manifest differently from classic PG, potentially contributing to diagnostic delay. These findings emphasize the importance of integrating clinical, histopathologic, and microbiological data for diagnosis.

The close temporal relationship between secukinumab initiation and lesion onset, followed by rapid pain improvement after corticosteroid therapy and progressive healing after drug discontinuation and cyclosporine initiation, strongly supported a probable paradoxical association.

## Conclusion

This case highlights a probable paradoxical pyoderma gangrenosum associated with secukinumab and reinforces the importance of clinicopathologic correlation in the diagnosis of biologic-associated neutrophilic dermatoses.

## Conflicts of interest

None disclosed.
